# Commentary: *SPTBN5*, encoding the βV-spectrin protein, leads to a syndrome of intellectual disability, developmental delay, and seizures

**DOI:** 10.3389/fnmol.2022.1011856

**Published:** 2022-09-02

**Authors:** Danique Beijer, Stephan L. Züchner

**Affiliations:** Dr. John T. Macdonald Foundation, Department of Human Genetics and John P. Hussman Institute for Human Genomics, University of Miami, Miller School of Medicine, Miami, FL, United States

**Keywords:** spectrin (α/β), monogenic disease, next generation sequencing (NGS), neurodevelopmental disorders, genetic data analysis, spectrin cytoskeleton

The spectrin-cytoskeleton, consisting of combinations of alpha-spectrin and beta-spectrin subunits, is essential for preserving the integrity and mechanical characteristics of the cell membrane. In the nervous system it is critical for the correct assembly and maintenance of neuronal excitable domains including the axon initial segment and the nodes of Ranvier (Liu and Rasband, 2019).

Dysfunction of the spectrin-cytoskeleton and their associated proteins has been implicated in several human diseases, but with a particular enrichment for neurodevelopmental and neurodegenerative diseases (Liu and Rasband, 2019). The non-erythroid variants of spectrin proteins (SPTAN1, SPTBN1,2,4 and 5) have now all been implicated in neurological diseases with either dominant or recessive inheritance, sometimes both (Ikeda et al., 2006; Lise et al., 2012; Knierim et al., 2017; Syrbe et al., 2017; Wang et al., 2018; Beijer et al., 2019; Rosenfeld et al., 2021; Khan et al., 2022; Van De Vondel et al., 2022).

One of the main challenges within the group of the spectrin gene mutations is the interpretation of candidate variants. All non-erythroid spectrin protein can be considered large containing at least 2,300 residues. Therefore, despite their general genetic constraint, spectrin genes still accumulate a large number of variants, of which only a small number will be causative for disease. In addition, the phenotype associated with spectrin mutations is rapidly expanding, which poses difficulties for the interpretation of these variants in the clinical context, resulting in many variants of unknown significance (VUS) (Savarese et al., 2020).

We read with great interest the newest paper in this field, which implicates four different heterozygous *de novo* variants in *SPTBN5* (His89Pro, Tyr311^*^, Asn2937Tyr, Glu3262Lys) as the disease cause in patients with a combination of intellectual disability, developmental delay and seizures (Khan et al., 2022). The study is appealing in the context of other spectrin-related diseases but raises some concerns that need to be addressed. In fact, there are two main limitations to this study, which combined raise the question of the true pathogenicity of the four variants implicated to cause the neurodevelopmental phenotype.

The first limitation is the general approach to filtering of the whole-exome sequencing (WES) where all variants with a minor allele frequency (MAF) <1% in the Exome Variant Server, GnomAD and 1000 Genomes were considered. While not necessarily wrong, even under an autosomal dominant hypothesis, further consideration was needed, when a heterozygous allele count of 24 and 1,042 (and 4 homozygous counts) was observed for the variants Glu3262Lys and Asn2937Tyr, respectively. These heterozygous allele counts are generally considered too high for fully penetrant dominant and rare disorders, especially considering the early-onset and severity of the disease described (Pipis et al., 2019). It is further surprising that these two variants would be observed *de novo* in the study, when they have already been observed not unfrequently in the population.

The second limitation of the study is the lack of a description of genetic testing performed to confirm the parentage of the affected patients. Especially for the variants with the increased allele count, the presence in the general population strongly supports a dominant inheritance rather than a *de novo* inheritance. However, even for the two variants His89Pro and Tyr311^*^, for which the heterozygous allele count in gnomAD could be supportive of pathogenicity (allele counts of 1 and 2 respectively), the confirmation of parentage is essential to conclude a *de novo* inheritance. It is finally unclear how a *de novo* variant would occur in two siblings in two of the presented families. Genetic mosaicism is a possibility, but was not mentioned by Khan et al., and appears unlikely to be present in two out of four reported families. Finally, the paper at the end of the Introduction mistakenly states a homozygous occurrence of these four variants in the patients, which is not supported by the provided figure and a later statement in the Discussion.

Overall, an association between *SPTBN5* and a neurodevelopmental phenotype would not be unexpected and would fit well with the overall importance of the spectrin-cytoskeleton in the nervous system. However, further evidence is needed for *SPTBN5* to join the growing list of monogenic disorders caused by spectrin genes.

## Author contributions

DB drafted the initial manuscript. DB and SZ revised the manuscript. All authors contributed to the article and approved the submitted version.

## Conflict of interest

The authors declare that the research was conducted in the absence of any commercial or financial relationships that could be construed as a potential conflict of interest.

## Publisher's note

All claims expressed in this article are solely those of the authors and do not necessarily represent those of their affiliated organizations, or those of the publisher, the editors and the reviewers. Any product that may be evaluated in this article, or claim that may be made by its manufacturer, is not guaranteed or endorsed by the publisher.
